# Therapeutic-dose heparin combined with antiplatelets in noncritically ill patients with COVID-19: a secondary analysis of a multiplatform randomized controlled trial

**DOI:** 10.1016/j.rpth.2025.102893

**Published:** 2025-05-21

**Authors:** Sylvain A. Lother, Wen Teng, Olawale Ayilara, Brett L. Houston, Barret Rush, Srinivas Murthy, Jose C. Nicolau, Lindsay Bond, Alexis F. Turgeon, John C. Marshall, Jonathan Paul, Judith S. Hochman, Matthew D. Neal, Michael E. Farkouh, Joel Nkosi, Donald S. Houston, Charlotte A. Bradbury, Asher A. Mendelson, Ewan C. Goligher, Allan Garland, Robert Balshaw, Souradet Y. Shaw, Patrick R. Lawler, Yoav Keynan, Ryan Zarychanski

**Affiliations:** 1 Department of Internal Medicine, Max Rady College of Medicine, University of Manitoba, Winnipeg, Manitoba, Canada; 2 Department of Medical Microbiology and Infectious Diseases, University of Manitoba, Winnipeg, Manitoba, Canada; 3 Division of Biostatistics, Child Health and Evaluative Sciences, The Hospital for Sick Children, Toronto, Ontario, Canada; 4 Department of Community Health Sciences, Max Rady College of Medicine, University of Manitoba, Winnipeg, Manitoba, Canada; 5 Department of Medical Oncology and Hematology, CancerCare Manitoba, Winnipeg, Manitoba, Canada; 6 Department of Pediatrics, University of British Columbia, Vancouver, British Columbia, Canada; 7 Departmento Cardio-pneumology, Instituto do Coracao, Faculdade de Medicina, Universidade de São Paulo, São Paulo, Brazil; 8 Ozmosis Research, Toronto, Ontario, Canada; 9 Department of Anesthesiology and Critical Care, Université Laval, Quebec City, Quebec, Canada; 10 Population Health and Optimal Health Practices Research Unit, Departments of Traumatology, Emergency Medicine, and Critical Care Medicine, Université Laval Research Center, Centre Hospitalier Universitaire de Quebec-Université Laval, Quebec City, Quebec, Canada; 11 Departments of Surgery and Critical Care Medicine, University of Toronto, Toronto, Ontario, Canada; 12 Department of Medicine, University of Chicago Medical Center, Chicago, Illinois, USA; 13 Division of Cardiology, Department of Medicine, Cardiovascular Clinical Research Center, NYU Grossman School of Medicine, New York, New York, USA; 14 Department of Surgery, Pittsburgh Trauma Research Center, University of Pittsburgh Medical Center, Pittsburgh, Pennsylvania, USA; 15 Departments of Academic Affairs and Cardiology, Cedars-Sinai Health System, Los Angeles, California, USA; 16 Department of Hematology, Faculty of Health Sciences, University of Bristol, Bristol, UK; 17 Interdepartmental Division of Critical Care Medicine, Department of Medicine and Physiology, University of Toronto, Toronto, Ontario, Canada; 18 George and Fay Yee Centre for Healthcare Innovation and Department of Community Health Sciences, University of Manitoba, Winnipeg, Manitoba, Canada; 19 Department of Medicine, McGill University Health Centre, McGill University, Montreal, Quebec, Canada; 20 Department of Medicine, University of Toronto, Toronto, Ontario, Canada

**Keywords:** aspirin, clopidogrel, COVID-19, heparin, SARS-CoV-2

## Abstract

**Background:**

Therapeutic-dose heparin improves outcomes in noncritically ill patients hospitalized for COVID-19. The effect of antiplatelet exposure in addition to therapeutic-dose heparin is unknown.

**Objectives:**

To evaluate the effect of antiplatelet exposure in addition to therapeutic-dose heparin on survival without organ support.

**Methods:**

We conducted an observational secondary analysis of a multiplatform randomized controlled trial, analyzing noncritically ill patients hospitalized for COVID-19 who received an antiplatelet agent (acetylsalicylic acid or P2Y12 inhibitor) and therapeutic-dose heparin (combination) compared with therapeutic-dose heparin alone (control). We used a 3-level ordinal primary outcome: (1) survival without organ support, (2) survival with organ support, and (3) mortality by day 21. Propensity scores were estimated using logistic regression. Balanced analytic groups were established using stabilized inverse probability of treatment weighting. A proportional odds model was used to estimate the effect of antiplatelet exposure.

**Results:**

Among 1021 patients, 194 (19.0%) were exposed to an antiplatelet (95.4% acetylsalicylic acid) and therapeutic-dose heparin. All patients were used to calculate the propensity scores and stabilized weights. After applying inverse probability of treatment weighting, the effective sample size was 60 in the combination group and 652 in the control group. Means and prevalences of continuous and dichotomous variables were similar between groups, with no evidence of misclassification. Exposure to an antiplatelet was not associated with improved survival without organ support (76.3% vs 80.5%; odds ratio, 1.07; 95% CI, 0.71-1.64).

**Conclusion:**

In noncritically ill patients hospitalized for COVID-19 receiving therapeutic-dose heparin, exposure to an antiplatelet agent was not associated with improved survival without organ support.

## Essentials

• Heparin is useful in noncritically ill COVID-19 patients, but the antiplatelet effect is unclear.
• We analyzed COVID-19 patients who received heparin with or without exposure to antiplatelets.
• Exposure to heparin and antiplatelets did not improve outcomes compared with heparin alone.
• Exposure to heparin and antiplatelets may increase bleeding risk, but bleeding events were rare.

## 1 Introduction

COVID-19 has increased global morbidity and mortality from respiratory infections since the onset of the pandemic [1]. Patients hospitalized for COVID-19 are at increased risk of micro- and macrovascular thrombosis and inflammation, leading to organ dysfunction and adverse clinical outcomes [[2], [3], [4], [5], [6]]. In COVID-19, interventions targeted at reducing host thrombotic and inflammatory responses have shown benefits, including anticoagulants and possibly antiplatelet agents [[7], [8], [9]]. The effectiveness of therapeutic-dose anticoagulation in combination with antiplatelet agents is uncertain.

In a previously reported multiplatform randomized controlled trial (mpRCT), which included the Antithrombotic Therapy to Ameliorate Complications of COVID-19 (ATTACC), A Multicenter Adaptive Randomized Controlled Platform Trial of the Safety and Efficacy of Antithrombotic Strategies in Hospitalized Adults with COVID-19 (ACTIV-4a), and Randomized Embedded Multifactorial Adaptive Platform Trial for Community Acquired Pneumonia (REMAP-CAP) platform trials, therapeutic-dose anticoagulation with heparin reduced organ dysfunction and improved survival in noncritically ill patients hospitalized for COVID-19 [7]. The benefit of therapeutic-dose heparin was replicated in subsequent randomized trials [[10], [11], [12], [13], [14], [15]], with decreased mortality in a prospective meta-analysis (11 trials, 6297 patients) [15]. Adjunctive therapeutic-dose heparin for the treatment of COVID-19 is now recommended or suggested for noncritically ill patients in many international guidelines [7,14,16,17].

When investigated as a single agent, antiplatelet agents have mixed effects in randomized controlled trials (RCTs) of patients hospitalized for COVID-19 [[18], [19], [20]]. Acetylsalicylic acid (ASA) or P2Y12 inhibitors failed to demonstrate clear benefit on hospital-based outcomes [18,20]; however, in an exploratory secondary analysis of a large, randomized trial, ASA improved survival at 180 days [8]. Combined anticoagulation and antiplatelet therapy are the cornerstone of therapy for thromboinflammatory conditions such as myocardial infarction [21]. The effect of combination therapy on outcomes for infections driving thromboinflammation has not been established.

To investigate whether the combination of therapeutic-dose heparin with an antiplatelet agent is associated with improved survival and reduced need for organ support compared with therapeutic-dose heparin alone, we conducted a propensity-weighted analysis of COVID-19 clinical trial data in the mpRCT.

## 2 Methods

### 2.1 Study design, setting, and population

We conducted an observational secondary analysis of noncritically ill participants enrolled in the ATTACC and ACTIV-4a trial platforms of the mpRCT [7,22,23]. The REMAP-CAP trial platform contributed 13% of participants in the mpRCT, but their data were not accessible for this secondary analysis. The mpRCT included noncritically ill patients hospitalized for COVID-19 between April 2020 and January 2021. Patients were randomly assigned to receive therapeutic-dose anticoagulation (with either low-molecular-weight heparin or unfractionated heparin) vs usual care venous thromboembolism prophylaxis. The platform trial protocols and data were federated and analyzed using a single statistical model, with detailed methods and findings reported in the mpRCT [7,22]. Since therapeutic-dose heparin was shown to improve the composite outcome of survival with reduced need for intensive care unit (ICU)-level organ support, we restricted our analysis to patients randomized to receive therapeutic-dose heparin.

### 2.2 Exposure groups

We defined exposure groups as: (1) patients who received therapeutic-dose heparin in combination with an antiplatelet agent (combination group) and (2) patients who received therapeutic-dose heparin alone (control group). Therapeutic-dose heparin was administered according to local practice, policies, and guidelines for the treatment of venous thromboembolism. Antiplatelet exposure was defined as receipt of at least 1 dose of ASA or a P2Y12 inhibitor (clopidogrel, prasugrel, or ticagrelor) during the first 14 days of hospitalization for COVID-19, which was not randomly allocated in the trial. We included patients receiving antiplatelet agents prehospitalization and those having new exposures.

### 2.3 Inclusion and exclusion criteria

Patients were included in this secondary analysis of the mpRCT if they were adults aged ≥18 years, admitted to hospital with laboratory-confirmed SARS-CoV-2, and had signs/symptoms compatible with COVID-19 disease. Patients were excluded if they received ICU-level organ support at the time of enrollment. ICU-level organ support was defined as the use of high-flow nasal oxygen, noninvasive ventilation (NIV), invasive mechanical ventilation (IMV), extracorporeal life support, vasopressors, and/or inotropes delivered in an ICU or repurposed critical care area. We also excluded patients who received 2 or more antiplatelet agents concomitantly due to bleeding risk.

### 2.4 Outcome measures

The primary outcome was a 3-level ordinal outcome reflecting survival to hospital discharge without ICU-level organ support (best outcome), survival to hospital discharge with ICU-level organ support (intermediate outcome), or all-cause mortality (worst outcome) in the first 21 days following randomization. We used organ support-free days (OSFD) to define these categories (OSFD 22 = no organ support; OSFD 0-21 = received ICU-level organ support; OSFD −1 = death). Secondary outcomes included survival without ICU-level respiratory support (including high-flow nasal oxygen, NIV, and IMV), survival without need for IMV, 90-day mortality, hospital-free days to day 28, and total thrombotic events (any systemic arterial embolization, stroke, myocardial infarction, deep vein thrombosis, or pulmonary embolism; Supplementary Table 1). Safety outcomes included major bleeding, as defined according to the International Society on Thrombosis and Haemostasis [24].

### 2.5 Baseline comparisons of exposure groups

We summarized baseline characteristics as means ± SDs or medians with IQRs for parametric and nonparametric continuous variables, respectively. Categorical variables were presented as proportions. Standardized mean differences were used to compare baseline characteristics between the combination and control groups [25].

### 2.6 Propensity score estimation

Propensity scores were used to estimate a patient’s probability of receiving an antiplatelet agent, given their baseline characteristics. We estimated the propensity score using multivariable logistic regression, where receipt of an antiplatelet agent was modeled as the outcome variable (Supplementary Table 2). Model covariates comprised patient characteristics that were plausibly related to the primary outcome or the administration of an antiplatelet agent. Age, platelet count, and creatinine were modeled as continuous variables; D-dimer was categorized as high (≥2× upper limit of normal) or low (<2× upper limit of normal) according to local laboratory criteria; respiratory support was categorized as no oxygen, receipt of low-flow oxygen, or receipt of high-flow oxygen at the time of randomization. Comorbid conditions were dichotomized as present or absent. Cardiovascular disease was defined as having at least 1 of the following baseline conditions: heart failure, coronary artery disease, peripheral arterial disease, or cerebrovascular disease (stroke or transient ischemic attack). Missing data for continuous variables were handled by imputing the median for missing data points or were included as dummy missing variables for ordinal covariates; missing dichotomous comorbidity variables were considered absent.

### 2.7 Methods to achieve balance between exposure groups

Overlap of propensity score distributions between the combination and control groups was analyzed by a histogram plot (Supplementary Figure 1). To adjust for imbalances, we converted propensity scores to inverse probability of treatment weights (IPTWs) and calculated stabilized IPTWs, applying these to each covariate. We used an iterative approach to specify the final treatment model through the addition of covariates and interaction terms and by modeling the relationship between continuous variables and treatment status using nonlinear terms. The final model output maximally balanced baseline characteristics between the combination and control groups. The ability of the model to balance covariates was evaluated using mean standardized differences (Table 1). An absolute standardized difference <0.1 was considered adequate covariate balance [26].

**Table 1:** Baseline characteristics of patients exposed and unexposed to an antiplatelet agent randomized to receive therapeutic-dose anticoagulation with heparin before and after stabilized inverse probability of treatment weighting.

Variable	Unweighted cohort			Weighted cohort poststabilized IPTW			
	TAC + APT (*n* = 194)	TAC alone (*n* = 827)	Standardized difference	TAC + APT (*n* = 60)^b^	TAC alone (*n* = 652)^b^	Standardized difference	% Reduction in absolute standardized difference
Age (y), mean (SD)	67.4 (11.6)	57.5 (14.0)	0.702^a^	62.0 (13.9)	59.7 (14.5)	0.163^a^	77
Female sex (%)	46.4	38.6	0.160^a^	37.8	39.1	−0.027	83
Race (%)							
White	52.1	54.7	−0.052	58.1	54.2	0.076	0
Asian	2.6	3.9	−0.069	1.2	3.3	−0.114^a^	0
Black	30.9	16.2	0.375^a^	18.8	18.7	0.001	100
Indigenous	1.5	12.2	−0.353^a^	1.6	1.0	−0.284^a^	20
Ethnicity (%)							
Hispanic or Latino	21.6	48.9	−0.548^a^	41.2	44.5	−0.067	88
BMI, mean (SD)	32.3 (7.8)	31.1 (7.7)	0.151^a^	31.8 (7.4)	31.2 (7.8)	0.081	46
Comorbidities (%)							
Immunosuppression	14.9	6.7	0.302^a^	7.9	8.2	−0.011	96
CVD	38.7	5.7	1.017^a^	15.3	13.3	0.061	94
Respiratory disease	27.3	15.7	0.302^a^	32.3	18.4	0.364^a^	0
Diabetes	51.0	24.9	0.571^a^	32.3	30.2	0.045	92
Hypertension	76.8	47.0	0.596^a^	52.9	53.1	−0.003	99
CKD	20.1	4.5	0.595^a^	9.0	8.9	0.004	99
Oxygen support (%)							
No oxygen	12.9	15.4	−0.069	23.6	15.3	0.233^a^	0
Low-flow oxygen	74.2	76.7	−0.057	67.9	76.2	−0.195^a^	0
High-flow oxygen	2.6	1.3	0.100^a^	1.1	1.4	−0.029	71
Cotreatments (%)							
Corticosteroids	10.3	5.0	0.226^a^	9.1	5.2	0.165^a^	27
Remdesivir	0.5	0.2	0.051	0.2	0.2	−0.006	88
Laboratory values, mean (SD)							
Creatinine (mg/dL)	1.4 (1.9)	1.0 (0.8)	0.341^a^	1.2 (1.3)	1.1 (1.0)	0.027	92
Platelets (×10^9^)	216 (80)	243 (103)	−0.272^a^	224 (81)	236 (102)	−0.124^a^	54
D-dimer (ULN)^c^	4.1 (8.5)	2.6 (4.3)	0.276^a^	2.7 (5.1)	2.7 (4.2)	−0.005	98
Country (%)							
Brazil	8.8	26.2	−0.416^a^	22.3	23.0	−0.017	96
Canada	11.9	9.2	0.090	7.6	9.4	−0.061	32
United States	77.3	47.3	0.602^a^	67.0	53.4	0.273^a^	55

APT, antiplatelet; BMI, body mass index; CKD, chronic kidney disease; CVD, cardiovascular disease; IPTW, inverse probability of treatment weighting; TAC, therapeutic-dose anticoagulation; ULN, upper limit of normal.

a Absolute standardized difference > 0.1.

b Effective sample size after weighting.

c D-dimer was measured as the ratio above the ULN according to local laboratory criteria.

### 2.8 Stabilized IPTW

Weights were calculated from propensity scores to create a pseudopopulation where the distribution of potentially confounding variables was balanced between the combination and control groups [27]. Each patient was assigned a weight equal to the inverse of their propensity score. To reduce the disproportionate influence of extreme propensity scores (scores approaching 0 in the combination group or scores approaching 1 in the control group), we adjusted individual weights with the *stabilized* IPTWs, which were standardized to the overall mean of the propensity score [28].

### 2.9 Statistical analysis

Baseline characteristics of the unweighted and weighted cohorts post*stabilized* IPTW were summarized as means (SD) for continuous variables and percentages for categorical variables. We used a proportional odds model to estimate the effect of exposure to an antiplatelet agent on the primary ordinal outcome. An odds ratio (OR) >1 represents greater odds of survival to hospital discharge without ICU-level organ support. We tested the proportional odds assumption using a likelihood ratio test. To mitigate potential residual confounding, *a priori* sensitivity analyses were planned and conducted by adding unbalanced covariates after weighting (SD > 0.1) to the proportional odds model. Effect estimates for secondary outcomes were calculated using binary logistic regression for dichotomous outcomes and a proportional odds model for ordinal outcomes. All analyses were conducted using R version 4.2.2 and the twang package [29]. Confidence limits and *P* values reported reflect an ***α*** of 0.05.

## 3 Results

### 3.1 Baseline characteristics of the unweighted cohort

We included 1021 noncritically ill patients from the mpRCT who were randomized to receive therapeutic-dose heparin (Figure). The proportion of these patients with concurrent antiplatelet exposure was 19% (*n* = 194). The most commonly used antiplatelet agent was ASA (*n* = 186; 95.4%). Patients who received therapeutic-dose heparin in combination with an antiplatelet agent were older (67.4 vs 57.5 years), more likely female (46.4% vs 38.6%), and more likely to have comorbidities such as cardiovascular disease (38.7% vs 5.7%), diabetes (51.0% vs 24.9%), and hypertension (76.8% vs 47.0%) compared with those who received therapeutic-dose heparin alone. Baseline covariates before and after weighting are reported in Table 1.

**Figure:**
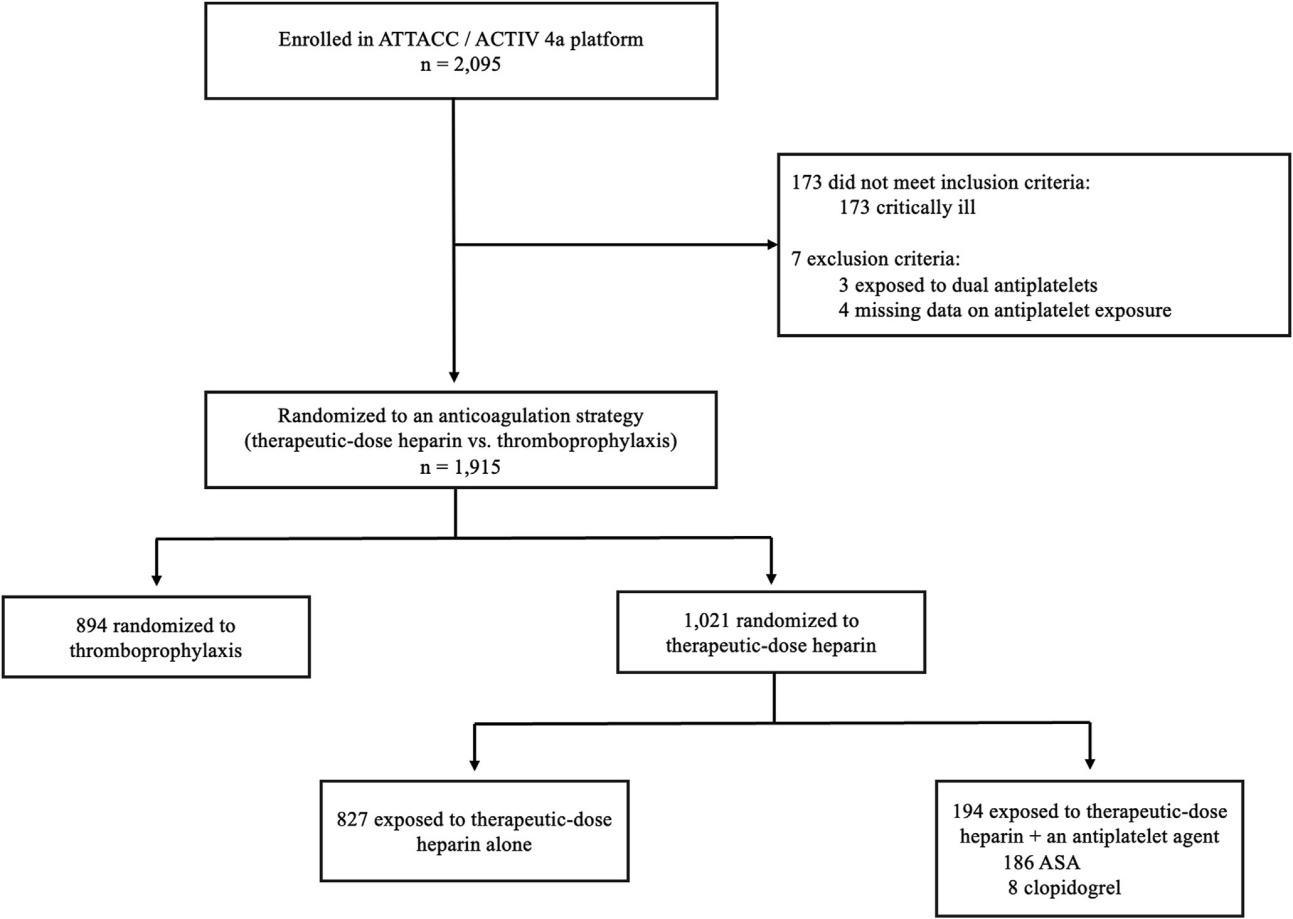
Study flow diagram. ACTIV 4a, A Multicenter Adaptive Randomized Controlled Platform Trial of the Safety and Efficacy of Antithrombotic Strategies in Hospitalized Adults with COVID-19; ASA, acetylsalicylic acid; ATTACC, Antithrombotic Therapy to Ameliorate Complications of COVID-19.

### 3.2 Comparison of baseline covariate distribution before and after propensity weighting and model diagnostics

Absolute standardized differences exceeded 0.1 in 76% of variables at baseline. After propensity weighting, covariate balance was improved between groups; residual standardized differences >0.1 were present in 36% of covariates (Table 1). In the weighted cohort, absolute standardized differences ranged from 0.001 to 0.364, with a median of 0.061 (IQR, 0.017-0.134), indicating that the means and prevalences of continuous and dichotomous variables were similar between groups (Supplementary Figure 2). The mean stabilized IPTW was 0.816 (SD, 1.220; range, 0.190-12.220) in the combination group and 1.009 (SD, 0.523; range, 0.810-7.580) in the control group, indicating no evidence of nonpositivity or misspecification of the propensity score model (Supplementary Figure 3).

### 3.3 Estimation of treatment effects

In patients receiving therapeutic-dose heparin, exposure to an antiplatelet agent was not associated with an improvement in survival without ICU-level organ support (OR, 1.07; 95% CI, 0.71-1.64; *P* = .76). In patients who received an antiplatelet agent in addition to therapeutic-dose heparin, 76.3% survived to hospital discharge without ICU-level organ support compared with 80.5% who received only therapeutic-dose heparin (Table 2). The proportional odds assumption was met using a likelihood ratio test (*P* = .075).

**Table 2:** The effect of exposure to an antiplatelet agent on secondary outcomes in patients receiving therapeutic-dose heparin after propensity weighting.

Primary outcome	TAC + APT	TAC alone	OR (95% CI)	*P* value
Survival to hospital discharge without ICU-level organ support (%)	76.3	80.5	1.07 (0.71-1.64)	.76
**Secondary outcomes**				
Survival to hospital discharge without ICU-level RS support (%)	83.1	84.8	1.58 (0.93-2.68)	.088
Survival to hospital discharge without the need for IMV (%)	75.3	80.0	0.35 (0.06-1.13)	.138
90-d mortality (%)	11.9	8.8	0.65 (0.33-1.19)	.19
Hospital-free days (d)	20.3	21.2	1.03 (0.77-1.38)	.857
Total thrombosis (%)	3.1	1.7	0.55 (0.09-1.95)	.43
Major bleeding events (%)	4.1	1.0	1.69 (0.43-5.19)	.39
Transfusion of ≥2 RBC units (%)	2.1	0.4	3.67 (0.59-19.13)	.12

APT, antiplatelet; ICU, intensive care unit; IMV, invasive mechanical ventilation; OR, odds ratio; RBC, red blood cell; RS, respiratory; TAC, therapeutic anticoagulation.

In sensitivity analyses, the addition of residual unbalanced covariates after propensity weighting yielded similar treatment effects, with no material differences in the magnitude of interpretation of the primary outcome (Supplementary Table 3).

### 3.4 Secondary outcomes

Exposure to an antiplatelet agent was not associated with statistically significant changes in major bleeding, transfusion of ≥2 red blood cell units, or any other secondary outcome (Table 2). Major bleeding in patients receiving therapeutic-dose heparin and an antiplatelet agent compared with those receiving therapeutic-dose heparin alone was 4.1% vs 1.0% (OR, 1.69; 95% CI, 0.43-5.19).

## 4 Discussion

In noncritically ill patients hospitalized for COVID-19 who were enrolled in the ATTACC and ACTIV-4a platforms of the mpRCT and randomized to receive therapeutic-dose heparin, inpatient exposure to an antiplatelet agent was not significantly associated with improved survival to hospital discharge without ICU-level organ support compared with treatment with therapeutic-dose heparin alone. While major bleeding events were uncommon, they were more frequent in patients who received both an antiplatelet agent and therapeutic-dose heparin, but were not statistically different and driven by the need for blood transfusion (Table 2).

Our findings are consistent with COVID-19 clinical trial evidence, which primarily shows no consistent benefit of the combination of an antiplatelet added to an anticoagulant. In the ACTIV-4a trial (*N* = 562), randomization of noncritically ill hospitalized patients to therapeutic-dose heparin plus a P2Y12 inhibitor vs therapeutic-dose heparin alone did not improve OSFD (OR, 0.83; 95% credible interval (CrI), 0.55-1.25; posterior probability of inferiority, 81%) [30]. Major bleeding occurred in 6 patients randomized to therapeutic-dose heparin plus a P2Y12 inhibitor vs 2 patients randomized to therapeutic-dose heparin alone [30]. In the antiplatelet domain of the REMAP-CAP trial, randomization to an antiplatelet agent (ASA or P2Y12) while concurrently receiving therapeutic-dose heparin (*n* = 122 critically ill patients with COVID-19) did not improve OSFD (OR, 0.73; 95% CrI, 0.44-1.21) or survival to hospital discharge (OR, 0.72; 95% CrI, 0.41-1.28) compared with low-dose thromboprophylaxis and no antiplatelet [18].

Therapeutic-dose heparin alone has consistently been shown to improve outcomes in RCTs of hospitalized noncritically ill patients with COVID-19 [7,[10], [11], [12],^31^]. In the mpRCT (*N* = 2219), therapeutic-dose heparin reduced the composite ordinal endpoint of progression to ICU-level organ support and death compared with usual care (OR, 1.27; 95% CrI, 1.03-1.58; posterior probability for superiority, 98.6%) [7]. Among critically ill patients, neither intermediate- nor therapeutic-dose heparin conferred clinical benefit [22]. Antiplatelet agents alone have shown mixed results in 4 RCTs of hospitalized COVID-19 patients [18,20,30,32]. The Randomised Evaluation of COVID-19 Therapy (RECOVERY) trial (*N* = 14,892) of patients receiving no or low-flow oxygen at baseline (63%) and those on higher-level respiratory support with NIV or IMV (33%) demonstrated no effect of ASA on 28-day mortality and no difference in the composite outcome of ventilation or death, but a significant yet small reduction in thrombotic events (4.6% vs 5.3%; absolute difference, −0.6%; SE, 0.4%) [20]. The REMAP-CAP (*N* = 1549) and Prevention of Arteriovenous Thrombotic Events in Critically-Ill COVID-19 Patients (COVID-PACT, *N* = 292) trials failed to demonstrate benefit with ASA or P2Y12 inhibitors alone [18,32]. In the REMAP-CAP platform trial, in 1549 critically ill patients, randomization to an antiplatelet agent had no impact on OSFD but improved hospital and 6-month survival compared with no antiplatelet (31.5% vs 32.4% 6-month mortality; hazard ratio, 0.85; 95% CrI, 0.71-1.03; 95% posterior probability of superiority) [8,18]. The practical implications of these findings have led to recommendations to use therapeutic-dose heparin in noncritically ill COVID-19 patients [14]. In contrast, use of antiplatelet agents alone and in combination with therapeutic-dose heparin is not recommended [17].

Given the benefit of therapeutic-dose heparin in COVID-19, this treatment is currently being investigated in non–COVID-19 pneumonia (NCT04372589). Antiplatelet agents may also be beneficial in non–COVID-19 pneumonia; however, the certainty of evidence is low [33]. While this study was post hoc in nature and remains hypothesis-generating, it suggests that the combination of therapeutic-dose heparin with an antiplatelet agent should be avoided in future trials of COVID-19 and non–COVID-19 pneumonia. However, this study does not provide sufficient evidence to suggest stopping or holding an antiplatelet agent when using therapeutic-dose heparin for the treatment of COVID-19.

Strengths of this study included the use of a robust, prospectively collected clinical trial dataset to evaluate patient baseline covariates, COVID-19 illness severity, treatment factors, and outcomes. We used a comprehensive propensity weighting analysis to reduce confounding and balance covariate distributions, including baseline cardiovascular disease and diabetes, which were significantly associated with exposure to an antiplatelet agent.

Our study had some limitations. First, we were unable to balance all covariates that might influence whether a patient receives an antiplatelet agent or the study’s primary outcome. Remaining between-group differences in covariate balance were most common in covariates that had a low prevalence (eg, Asian and Indigenous race, use of corticosteroids, and no oxygen support at baseline), and thus unlikely to significantly impact the results. Although some covariates remained unbalanced after weighting, we conducted sensitivity analyses to account for potential residual confounding, which yielded similar effect estimates. Second, while we included >1000 patients in the analysis, most patients did not receive an antiplatelet agent (81%), reducing the effective sample size after weighting and limiting the power to detect between-group differences. Third, the trial dataset was limited to shorter-term outcomes (organ support to 21 days; mortality to 90 days), limiting our ability to test the hypothesis that the addition of antiplatelet agents could be beneficial with longer-term follow-up (such as 180 days), as demonstrated in the REMAP-CAP trial of antiplatelet agents [8]. Fourth, the duration of exposure to an antiplatelet agent and proportion of patients on antiplatelets prior to COVID-19 illness were not recorded in our dataset; thus, the effect of exposure duration and indication for antiplatelet use is unknown. It is possible that duration of exposure and follow-up duration was too short to observe a treatment effect in COVID-19. Fifth, our analysis was limited to patients with COVID-19 pneumonia early in the pandemic, prior to the emergence of the Delta and Omicron variants, and prior to widespread availability of vaccines, population-level immunity, and widespread use of corticosteroids. Given the results of this study, which found no association with the outcome, these factors are unlikely to influence current practice. Finally, little is known about the effects of therapeutic-dose heparin or antiplatelet agents on other non–COVID-19 causes of pneumonia.

## 5 Conclusion

In noncritically ill patients hospitalized for COVID-19 receiving therapeutic-dose heparin, exposure to an antiplatelet agent was not associated with improved survival without ICU-level organ support.
