# Supplementary material for: Therapeutic-dose heparin combined with antiplatelets in noncritically ill patients with COVID-19: a secondary analysis of a multiplatform randomized controlled trial
Source: Res Pract Thromb Haemost. 2025 May 21;9(4):102893. doi: 10.1016/j.rpth.2025.102893 (PMC12226087; doi:10.1016/j.rpth.2025.102893)
Supplement: Supplementary Material [file mmc1.docx]

**Supplementary Material**

**Supplementary Table 1**: Description of primary and secondary outcome measures

| **#** | **Endpoint** | **Variable type** | **Description** |
| --- | --- | --- | --- |
| 1.0 | Survival without ICU-level organ support | Ordinal | 3-level ordinal outcome based on the worst status of each patient through day 21   1. Survival without ICU-level organ support 2. Survival with ICU-level organ support 3. Death |
| 2.1 | Survival without ICU-level respiratory support | Ordinal | 3-level ordinal outcome based on the worst status of each patient through day 21   1. Survival without HFNO, NIMV or IVM (without respiratory support) 2. Survival with HFNO, NIV or IMV 3. Death |
| 2.2 | Survival without need for IMV | Ordinal | 3-level outcome based on worst status of each patient through day 21   1. Survival without IMV 2. Survival with IMV 3. IMV and/or death |
| 2.3 | 90-day mortality | Dichotomous | Dichotomous outcome measured at day 90   1. Survival 2. Death |
| 2.4 | Hospital-free days | Ordinal | Number of days not admitted to hospital within the first 21 days (range -1 to 21, where -1 indicates death) |
| 2.5 | Total thrombotic events | Dichotomous | Dichotomous outcome measured through day 21   1. Thrombotic event (MI, stroke, arterial embolization, DVT, PE) 2. No thrombotic event |
| 2.6 | Major bleeding | Dichotomous | Dichotomous outcome measured through day 14   1. Major bleeding event 2. No major bleeding event |
| 2.7 | Transfusion ≥ 2 units pRBC | Dichotomous | Dichotomous outcome measured through day 14   1. Received ≥ 2 units pRBC 2. Did not receive ≥ 2 units pRBC |

DVT = deep venous thrombosis; HFNO = high flow nasal oxygen; ICU = intensive care unit; IMV = invasive mechanical ventilation; MI = myocardial infarction; NIV = non-invasive ventilation; PE = pulmonary embolism; pRBC = packed red blood cells

**Supplementary Table 2**: Logistic regression to evaluate the association of covariates on exposure to an antiplatelet agent

| **Covariate** | **Description** | **Odds ratio** | **95% CI** |
| --- | --- | --- | --- |
| Sex | Male | Ref | |
|  | Female | 1.14 | 0.75 – 1.73 |
| Race | White | Ref | |
|  | Asian | 0.95 | 0.27 – 3.32 |
|  | Black | 1.38 | 0.82 – 2.33 |
|  | First Nations | 0.31 | 0.06 – 1.59 |
|  | Other | 0.90 | 0.19 – 4.31 |
|  | Unknown | 2.28 | 1.03 – 5.04 |
| Ethnicity | Not Hispanic/Latino | Ref | |
|  | Hispanic/Latino | 0.68 | 0.33 – 1.39 |
|  | Unknown | 0.44 | 0.12 – 1.70 |
| BMI | < 18.5 | Ref | |
|  | 18.5 – 24.9 | 0.33 | 0.07 – 1.59 |
|  | 25 – 29.9 | 0.63 | 0.14 – 2.91 |
|  | 30 – 34.9 | 0.88 | 0.19 – 4.14 |
|  | 35 – 39.9 | 0.68 | 0.14 – 3.42 |
|  | > 40 | 0.81 | 0.16 – 4.00 |
|  | Unknown | 0.37 | 0.07 – 1.86 |
| Immunosuppression | Dichotomous | 1.53 | 0.82 – 2.87 |
| Cardiovascular disease | Dichotomous | 6.11 | 3.74 – 9.98 |
| Respiratory disease | Dichotomous | 1.09 | 0.68 – 1.75 |
| Diabetes | Dichotomous | 2.09 | 1.38 – 3.17 |
| Hypertension | Dichotomous | 1.32 | 0.83 – 2.09 |
| CKD | Dichotomous | 1.42 | 0.73 – 2.77 |
| Liver disease | Dichotomous | 0.99 | 0.23 – 4.20 |
| Respiratory support | No O2 | Ref | |
|  | Low-flow O2 | 1.00 | 0.53 – 1.88 |
|  | High-flow O2 | 0.72 | 0.15 – 3.43 |
|  | Unknown | 1.13 | 0.46 – 2.80 |
| Creatinine | Continuous variable* | 1.06 | 0.90 – 1.24 |
| Platelet count | Continuous variable* | 1.00 | 1.00 – 1.00 |
| D-dimer | Low | Ref | |
|  | High | 1.54 | 0.98 – 2.41 |
|  | Unknown | 1.10 | 0.61 – 1.97 |
| Corticosteroid treatment | Dichotomous | 1.88 | 1.18 – 2.97 |
| Remdesivir treatment | Dichotomous | 2.18 | 0.16 – 29.06 |
| Country of enrolment | Canada | Ref | |
|  | Brazil | 0.42 | 0.14 – 1.26 |
|  | USA | 0.97 | 0.47 – 1.97 |

BMI = body mass index; CI = confidence interval; CKD = chronic kidney disease; Ref = reference value

For all dichotomous outcomes, the reference value was absence of the condition

*Odds ratio calculated for every 1 unit rise

**Supplementary Table 3**: Effect estimates for the primary outcome (survival to hospital discharge without ICU-level organ support) using different propensity analytic methods

| **Sensitivity analysis model** | **OR** | **95% CI** |
| --- | --- | --- |
| Primary model: APT exposure only | 1.07 | 0.71 – 1.64 |
| Sensitivity analysis: APT exposure and age covariates in the model | 1.12 | 0.74 – 1.72 |
| Sensitivity analysis: APT exposure, age, respiratory disease, and receipt of corticosteroids in the model* | 1.12 | 0.74 – 1.73 |

APT = antiplatelet; CI = confidence interval; IPTW = inverse probability of treatment weighting; OR = odds ratio; TAC = therapeutic anticoagulation

*Addition of imbalanced covariates after weighting (SD > 0.1) in the primary model to adjust for potential residual confounding

**Supplementary Figure 1**: Histogram distribution of propensity scores in both the combination group and control group


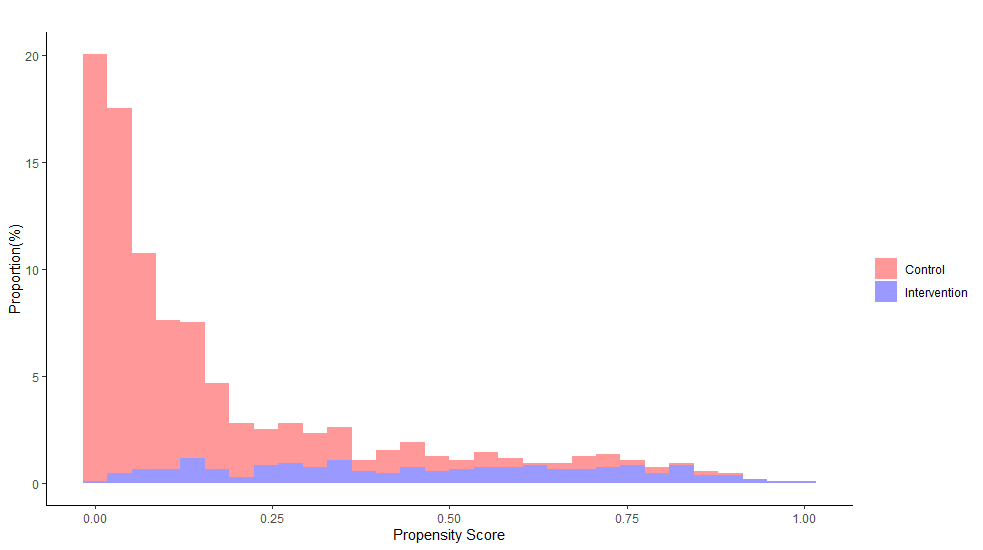


The propensity score indicates a patient’s propensity to be exposed to an antiplatelet agent given their observed characteristics. This histogram indicates a relatively even distribution of propensity scores in the combination group (patients who received an antiplatelet agent), however, it is heavily skewed to the left in the control group, suggesting these patients were highly unlikely to receive an antiplatelet agent given their observed characteristics. Given the imbalance between groups and extreme low weights in the control group, a decision was made to use stabilized inverse probability of treatment weighting to improve the balance between groups.

**Supplementary Figure 2**: Covariate balance measured by standardized difference in the unweighted and weighted cohorts


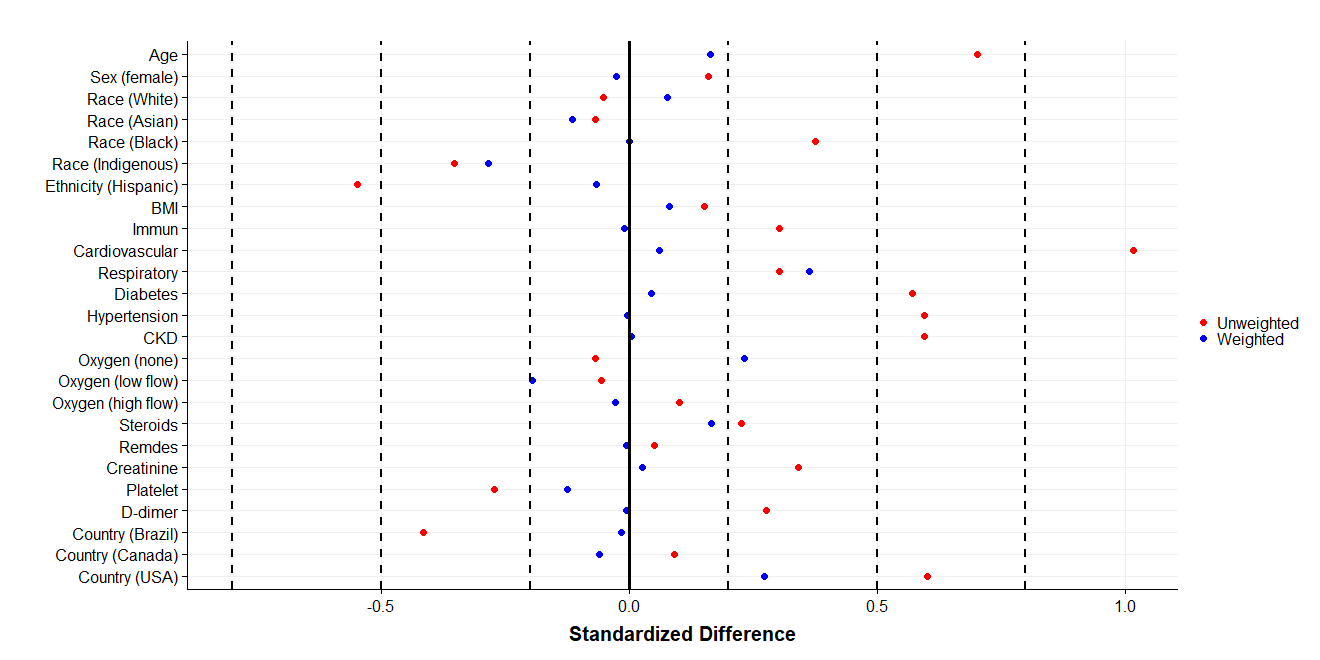


BMI = body mass index; CKD = chronic kidney disease; Immun = immunocompromised; Remdes = remdesivir

Standardized differences were used to compare the balance of measured variables between groups. Standardized differences < 0.1 are generally considered to be negligible (well balanced), standardized differences of 0.1 – 0.2 are considered to have possible small differences, 0.2 – 0.5 small differences, 0.5 – 0.8 medium differences, and > 0.8 large differences. The solid black lines indicate the threshold of well-balanced covariates, the dashed lines indicate possible but small differences in covariate balance. Many of the covariates in the unweighted cohort (red dots) have medium to high differences in the distribution of that covariate between the combination and control group. After weighting (blue dots), considerable improvement in the balance of covariates is observed between groups, with the majority achieving adequate balance from the weighting procedures.

**Supplementary Figure 3**: Cloud-plot of the distribution of stabilized inverse probability of treatment weighting scores in the combination and control groups


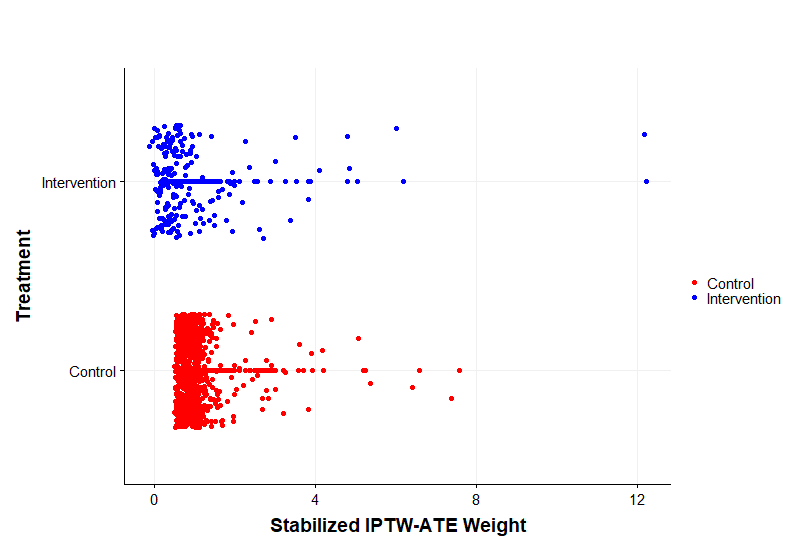


This cloud-plot demonstrates the distribution of propensity scores after stabilized inverse probability of treatment weighting (IPTW). This plot is generated to perform diagnostics on the propensity score model by evaluating the mean, standard deviation and range of scores between groups. If the mean of stabilized weights is far from 1.0, or if there are very extreme values (> 10), then this may indicate non-positivity or misspecification of the propensity score model. Iterative construction of propensity score models were constructed, and the final model was selected based on optimal diagnostic characteristics. In this cloud plot, the mean stabilized IPTW was 0.816 (SD 1.220, range 0.190 – 12.220) in the combination group and 1.009 (SD 0.523, range 0.810 – 7.580) in the control group indicating no evidence of non-positivity or misspecification of the propensity score model.
